# Expanding the scope of *Mycobacterium abscessus* reference strains to improve pulmonary disease modeling

**DOI:** 10.1242/dmm.052759

**Published:** 2026-09-01

**Authors:** Ruth A. Howe, Chavis Tabor, Chandra M. Panthi, Guoqing Cheng, Binayak Rimal, Evan Chen, Ngan Nguyen, Gyanu Lamichhane

**Affiliations:** ^1^ Division of Infectious Diseases, Department of Medicine, School of Medicine, Johns Hopkins University, Baltimore, MD 21287, USA; ^2^ Center for Nontuberculous Mycobacteria and Bronchiectasis, School of Medicine, Johns Hopkins University, Baltimore, MD 21287, USA

**Keywords:** *Mycobacterium abscessus*, *Mycobacteroides abscessus*, Reference strain, Lung infection, Mouse model

## Abstract

*Mycobacterium abscessus* (*Mab*) pulmonary disease is an emerging clinical challenge, particularly in individuals with immunosuppression or structural lung disease. No US Food and Drug Administration (FDA)-approved therapies exist, and current multidrug regimens are prolonged, toxic and achieve low cure rates, highlighting the need for improved treatments. Progress depends on preclinical models that faithfully recapitulate human disease, for which strain selection is critical. The widely used reference strain, ATCC 19977, was isolated from a non-pulmonary source but became the default owing to early availability. To assess its relevance for modeling pulmonary disease, we compared it with 15 clinical *Mab* isolates from lung infections across the United States. *In vitro* and in a validated mouse lung infection model, ATCC 19977 differed markedly from clinical isolates, exhibiting rapid systemic dissemination, limited lung granuloma formation and early mortality. In contrast, clinical isolates demonstrated greater pulmonary tropism, reduced dissemination and robust lung pathology. We propose a panel of pulmonary clinical isolates representing major *Mab* subspecies and phenotypes for lung research. These isolates more accurately recapitulate human disease and are expected to enhance the translational relevance of mechanistic and therapeutic studies.

## Teaser

**Summary:** Using a mouse model, this study identified *Mycobacterium abscessus* clinical isolates with infection profiles more closely resembling human pulmonary disease than ATCC 19977, establishing them as superior reference strains to ATCC 19977 for future translational and therapeutic research.

## INTRODUCTION

### Why *Mycobacterium abscessus* matters

Nontuberculous mycobacteria (NTM) are ubiquitous environmental organisms that cause opportunistic infections in immunocompetent and immunocompromised individuals (Falkinham, 2022; Johansen et al., 2020). Pulmonary NTM disease is of increasing clinical concern, particularly for patients with immune suppression, structural lung damage, cystic fibrosis and impaired mucociliary clearance. Its rising incidence exceeds what can be explained by improved detection alone, suggesting changes in exposure or a growing population of susceptible hosts (Cristancho-Rojas et al., 2024; Victoria et al., 2021).

Among NTMs, *Mycobacterium abscessus* (*Mab*) is the second most common cause of pulmonary disease after *Mycobacterium avium* and is notoriously difficult to treat, nicknamed ‘the clinical nightmare’ (Lopeman et al., 2019; Nessar et al., 2012). A recent study reclassified *Mycobacterium abscessus* as *Mycobacteroides abscessus* owing to distinct genomic features (Gupta et al., 2018). *Mab* often persists in patients through multiple antibiotic courses for other conditions, eventually transitioning from colonizing bystander to active pathogen (Benwill and Wallace, 2014; Kwak et al., 2019). Thus, *Mab* infections are frequently antibiotic experienced and resistant to multiple antimicrobial classes, and require prolonged multidrug regimens guided by expert opinion (Daley et al., 2020; Recchia et al., 2023). Fewer than half of patients achieve culture conversion, and therapies are expensive, toxic and poorly tolerated (Kwak et al., 2019; Park et al., 2017). The lack of any US Food and Drug Administration (FDA)-approved *Mab* treatment, coupled with poor clinical outcomes, underscores the urgent need for new, more effective and tolerable therapies.

### Why accurate disease models matter

Elucidation of *Mab* biology and disease pathogenesis depends on preclinical models that accurately reproduce human disease, as does therapeutic development. When laboratory models fail to mirror the clinical scenario, they can yield partial or misleading insights that impede our ability to understand disease pathogenesis and develop treatments. A well-known example comes from *Escherichia coli*: the common laboratory strain K-12 lacks the virulence plasmids, pathogenicity islands and secretion systems of pathogenic strains such as enterohemorrhagic *E. coli* O157:H7 (Perna et al., 2001). Consequently, results from K-12 often fail to predict how pathogenic *E. coli* behave in humans. Similar limitations in laboratory models have challenged research in infectious disease, oncology, neuroscience and other fields, illustrating a key principle: model systems must reflect the biology of human disease. As fields mature, it is common for multiple models to be developed to reflect specific disease scenarios and improve therapeutic development.

### Why *Mab* disease modeling is complicated

*Mab* disease can result from multiple routes of infection that lead to distinct disease outcomes. Direct inoculation through trauma or contaminated surgical equipment causes localized abscesses. In contrast, pulmonary infections result primarily from inhalation of aerosolized *Mab* originating from biofilm reservoirs in domestic plumbing and aqueous environments (Norton et al., 2020; Ruis et al., 2021; Thomson et al., 2025; Virdi et al., 2021).

The pulmonary versus inoculation infection routes expose *Mab* to divergent host environments and evolutionary pressures. Direct inoculation produces aggressive, abscess-forming infections even in immunocompetent individuals, whereas pulmonary infection typically begins as a chronic colonization and gradually progresses to granulomatous disease, particularly in individuals with immunocompromise or underlying structural lung abnormalities (Lee et al., 2015). Both routes can, occasionally, lead to systemic dissemination, producing diverse bacterial subpopulations with differing drug susceptibilities (Park et al., 2023). Whether dissemination depends more on pathogen or host factors remains uncertain. It remains unclear whether a single environmental or abscess-derived strain can naturally cause both forms of disease without additional adaptation. Consequently, strains isolated from non-pulmonary sources may not faithfully represent pulmonary pathogenesis.

For *Mab*, most laboratory studies have relied on the ATCC 19977 strain, originally isolated in 1950 from a knee abscess thought to be from traumatic inoculation of soil (Moore and Frerichs, 1953). Historical descriptions of the case noted no lung disease, and some apparent dissemination may have resulted from clinical practices of the time rather than hematogenous spread. Because ATCC 19977 was the first, and for decades only, *Mab* strain available from the American Type Culture Collection, it became the de facto reference strain for all studies. However, most *Mab* infections today are pulmonary, raising concern whether a strain derived from a knee abscess accurately models respiratory disease pathogenesis, host response or treatment response (Dartois et al., 2024).

Adding further complexity, *Mab* comprises at least two dominant subspecies, *Mycobacterium abscessus* and *Mycobacterium massiliense*, that differ in drug susceptibility due to Erm(41) activity (Tortoli et al., 2016). A third subspecies, *Mycobacterium massiliense bolettii*, has not yet reached clinical prominence, and additional environmental variants may exist. Differences in subspecies, colony morphotypes, and pulmonary versus extrapulmonary infection source all influence disease behavior and should be reflected in model selection (López-Roa et al., 2022). No single isolate will represent the full spectrum of clinical disease.

### Significance of this study

We asked whether ATCC 19977, recovered from a knee abscess over 75 years ago and laboratory adapted, adequately represents the *Mab* strains causing today's pulmonary infections. To address this, we compared ATCC 19977 with *Mab* isolates obtained from patients with lung disease caused by both major subspecies and originating from distinct geographical locations. Using *in vitro* assays and a validated mouse model of aerosol infection, we identified isolates that reproduced key features of human *Mab* pulmonary disease. These isolates more accurately reflect current clinical infections, and a subset are proposed as improved reference strains for future pathogenesis and therapeutic studies.

## RESULTS

### Subspecies and morphotype

*Mab* strains of subspecies *abscessus* and *massiliense* isolated from the lungs of patients from distinct regions of the United States were included in this study (Table 1). Phylogenetic analysis showed that these isolates did not cluster purely by geography and showed comparable diversity to strains previously published from the National Institutes of Health and multiple studies in Southeast Asia (Jin et al., 2022; Nakanaga et al., 2014; Ngeow et al., 2012) (Fig. S1).

**Table 1.** *Mab* pulmonary clinical isolates, subspecies, sourcing and growth phenotypes

Isolate ID	Subspecies	Source	Appearance on agar	Appearance in broth	Composite morphotype
M9502	*massiliense*	JHH	Mixed	Planktonic	Mixed
M9503	*abscessus*	JHH	Smooth	Planktonic	Smooth
M9507	*abscessus*	JHH	Smooth	Planktonic	Smooth
M9508	*abscessus*	JHH	Rough	Planktonic, heavy aggregate and heavy biofilm	Rough
M9510	*massiliense*	JHH	Smooth	Planktonic	Smooth
M9533	*abscessus*	JHH	Smooth	Planktonic	Smooth
M9624 CF00138	*abscessus*	NJH	Smooth	Planktonic	Smooth
M9626 CF00217	*abscessus*	NJH	Mixed	Planktonic, minimal aggregate and biofilm	Mixed
M9643 CF03548	*abscessus*	NJH	Mixed	Planktonic	Mixed
M9647 CF04462	*massiliense*	NJH	Rough	Planktonic, heavy aggregate and heavy biofilm	Rough
M9654 NC00036	*abscessus*	NJH	Smooth	Planktonic	Smooth
M9656 NC00041	*abscessus*	NJH	Smooth	Planktonic	Smooth
M9660 NC00049	*abscessus*	NJH	Mixed	Planktonic, heavy aggregate and biofilm	Rough
M9672 NC00272	*abscessus*	NJH	Mixed	Planktonic and biofilm	Mixed
M9674 NC00275	*abscessus*	NJH	Smooth	Planktonic	Smooth

NJH reference IDs are listed beneath their JHH reference ID, where applicable. ‘Appearance on agar’ refers to colony morphology on solid media (Fig. S4). ‘Appearance in broth’ refers to behavior in liquid media (Figs S2 and S3). ‘Composite morphotype’ refers to overall behavior across media types. JHH, Johns Hopkins Hospital, Baltimore, MD, USA; *Mab*, *Mycobacterium abscessus*; NJH, National Jewish Health, Denver, CO, USA.

One *in vitro* phenotype relevant to *Mab* pathogenesis is its ability to form biofilms (Greendyke and Byrd, 2008). We hypothesized that the container porosity influences *Mab* biofilm-forming capacity. To test this range for each isolate, we cultured *Mab* isolates in tubes made of materials with differing porosities with and without detergent. Under these conditions, aggressive biofilm formers would be expected to overcome inhibition from Tween-80 to aggregate or attach to substratum, while intermediate biofilm formers may only do so in the absence of detergent.

In high-porosity materials such as polypropylene and polystyrene, *Mab* isolates exhibited a range of growth behaviors, from forming prominent meniscal biofilms to entirely planktonic cultures (Table 1; Fig. S2). Some isolates also demonstrated a wall-climbing phenotype, establishing discrete colonies along the tube walls. In contrast, in high-density, low-porosity borosilicate glass tubes, growth ranged from pelleted aggregates settling at the bottom of the tubes to fully planktonic forms dispersed homogeneously throughout the broth. Although variable meniscal films were observed, none displayed climbing behavior. When detergent was removed from cultures in glass tubes, the meniscal films and aggregation behaviors became more pronounced, but no climbing behavior emerged (Fig. S3).

Another *in vitro* phenotype relevant to pathogenesis is colony morphotype on agar media. *Mab* colonies are classically described as either ‘smooth’, featuring glabrous domes with circular margins, or ‘rough’, characterized by matte, irregular and flaky colonies (Johansen et al., 2020). Although intermediate forms are not traditionally recognized, morphotype switching during infection has been documented (Byrd and Lyons, 1999; Howard et al., 2006). ATCC 19977 colony morphology was reported as mostly smooth with rare rough variants (Moore and Frerichs, 1953) and maintained that variable morphology in our hands. In contrast, clinical isolates consistently displayed uniform colony morphologies across a plate but rarely conformed to the classical extremes. Most exhibited smooth or mixed characteristics (Fig. S4). For clarity and comparison within the traditional smooth–rough framework, we categorized each strain as smooth, rough or mixed based on its behavioral range in liquid and solid media (Table 1).

### Drug susceptibilities

Numerous cellular processes contribute to antibiotic susceptibility and resistance, making minimum inhibitory concentration (MIC) panels informative snapshots of *Mab* physiologic state. We determined the MICs of 24 antibiotics spanning agents currently used in clinical treatment and compounds in preclinical development against all *Mab* isolates in this study (Table S1). As anticipated for clinical isolates collected at different stages of infection and treatment, MICs for commonly used antibiotics varied considerably. Many isolates exhibited resistance to macrolides. A subset displayed near pan-resistance across drug classes. These MIC patterns not only highlight the phenotypic diversity of *Mab* but also offer a functional framework for probing the underlying physiological and adaptive mechanisms that shape antibiotic susceptibility. *Mab* is notorious for discordance between *in vitro* MIC and *in vivo* efficacy, postulated to be mediated by limitations from slow growth relative to antimicrobial degradation *in vitro* and *in vivo* organization into protective biofilms (van Ingen and Kuijper, 2014). This poor concordance underlies the Clinical and Laboratory Standards Institute (CLSI) recommendation against performing MICs for non-macrolides to inform *Mab* treatment. In this study, strain morphotypes did not directly correlate with antibiotic susceptibilities *in vitro* for any agent. However, the correspondence of these morphotypes to biofilm formation or other structural organization *in vivo* may impact antibiotic susceptibilities and will require additional study.

### *In vivo* characteristics of ATCC 19977

A mouse model of *Mab* infection provides a controlled, tractable system for assessing the virulence potential of clinical isolates (Dartois et al., 2024). Here, virulence is defined by the ability of an isolate to establish and sustain pulmonary infection, trigger host immune responses, disseminate to other organs and induce pathological changes. By comparing these parameters using an identical protocol with *Mab* isolates as the only variable, distinct virulence patterns can be discerned, enabling the identification of *Mab* strains for which *in vivo* behavior profiles typify those observed in human *Mab* infections.

**Fig. 1.**
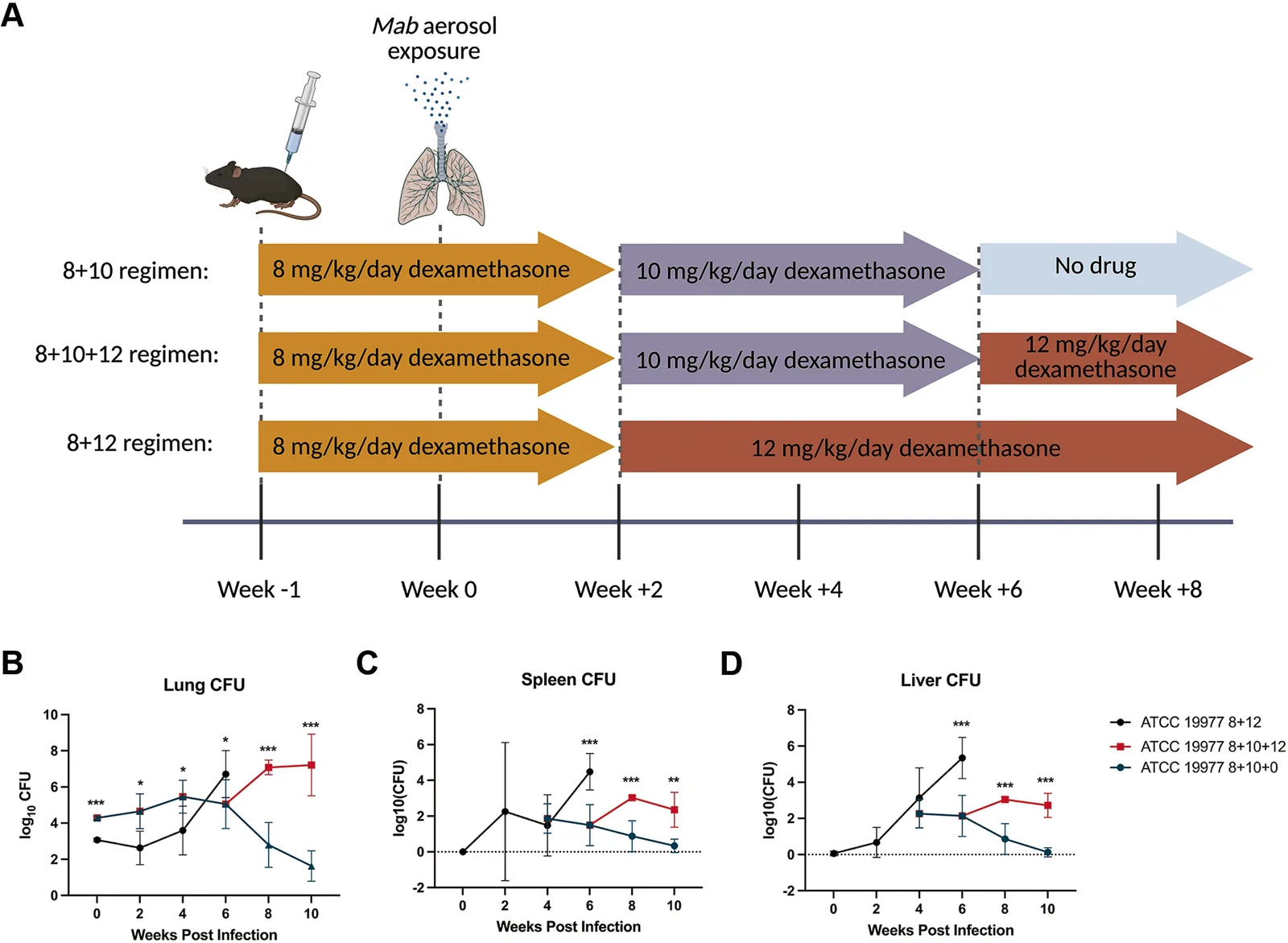
**Immunosuppression of C3HeB/FeJ mice and dynamics of infection with ATCC 19977.** (A) Schematic of immunosuppression and infection regimens used to determine activity of the ATCC 19977 reference strain in C3HeB/FeJ mice. (B–D) *Mycobacterium abscessus* (*Mab*) burden determined by colony-forming unit (CFU) assay in lung (B), liver (C) and spleen (D) in mice infected with ATCC 19977 under each dexamethasone (immunosuppressive scheme). Teal shapes indicate mice receiving 3 weeks of 8 mg/kg/day dexamethasone followed by 4 weeks of 10 mg/kg/day dexamethasone. Red squares indicate mice receiving 3 weeks of 8 mg/kg/day dexamethasone followed by 4 weeks of 10 mg/kg/day dexamethasone, followed by 12 mg/kg/day dexamethasone until death. Black circles indicate animals receiving 3 weeks of 8 mg/kg/day dexamethasone followed by 12 mg/kg/day dexamethasone until death. *P*-values by unpaired multiple *t*-testing with Welch correction, denoted by *P<0.05, **P<0.01 or ***P<0.001. Created in BioRender by Howe, R. (2026). https://BioRender.com/6r54bp7. This figure was sublicensed under CC-BY 4.0 terms.

We used a validated mouse model of *Mab* lung infection (Galanis et al., 2022; Maggioncalda et al., 2020) to characterize *in vivo* growth and dissemination of the isolates. To assess the chronic phase of infection, we extended the infection period to 8 weeks. C3HeB/FeJ (Kramnik) mice were infected with aerosolized ATCC 19977 and subjected to dexamethasone-based immunosuppression regimens as described in the original model protocol (Maggioncalda et al., 2020) (Fig. 1A). Although a daily dose of 8 mg/kg dexamethasone was sufficient to sustain infection during the early phase, this dose failed to promote *Mab* persistence and growth through the 8-week period. Therefore, the dexamethasone dose was increased to 10 mg/kg after week 2 post-infection to support extended infection. Under a regimen of 10 mg/kg/day dexamethasone, ATCC 19977 clearance from the lungs began at 4 weeks post-infection, with complete clearance observed after immunosuppression withdrawal at 6 weeks (8+10+0 regimen; Fig. 1B–D) prompting adjustment of dexamethasone dosing. Increasing dexamethasone dose to 12 mg/kg/day at 6 weeks (8+10+12 regimen) allowed ATCC 19977 to maintain a higher lung bacterial burden with stable dissemination to the spleen and liver.

**Fig. 2.**
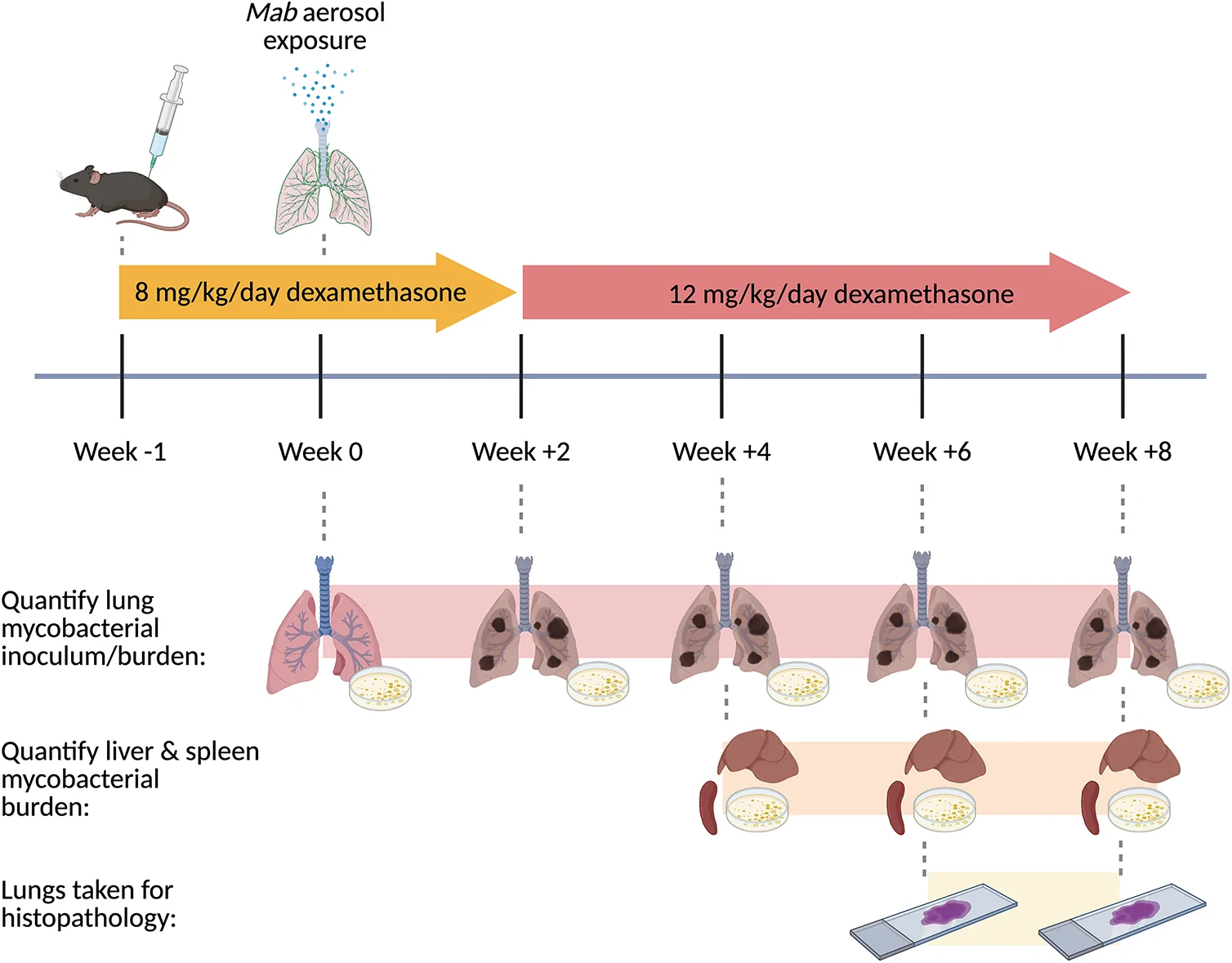
**Schematic of methods for immunosuppression, infection with ATCC 19977 and clinical pulmonary *Mab* isolates, and infection dynamics in C3HeB/FeJ mice.** Created in BioRender by Howe, R. (2026). https://BioRender.com/s5v35vi. This figure was sublicensed under CC-BY 4.0 terms.

However, clinical *Mab* isolates could not sustain pulmonary infection beyond 4 weeks under either 8+10+0 or 8+10+12 dexamethasone regimens. These isolates required more intensive immunosuppression, 8 mg/kg/day dexamethasone for the first 2 weeks post-infection followed by 12 mg/kg/day (8+12 regimen), to maintain or increase pulmonary bacterial loads (Fig. 2). To provide a valid comparator, we also profiled ATCC 19977 using the same 8+12 regimen (Fig. 1B–D). Under these conditions, ATCC 19977 caused early, aggressive systemic dissemination. Animals with lower pulmonary bacterial burdens succumbed early, exhibiting widespread microabscesses upon necropsy. All animals died before the 8-week endpoint, although histopathological analysis revealed *Mab*-positive lesions or scarring in only 50% of lungs examined (Table S2), suggesting that mortality was frequently due to extrapulmonary complications rather than primary pulmonary pathology.

### *In vivo* characteristics of pulmonary clinical *Mab* isolates

#### Lung-derived isolates produce greater pulmonary disease burden than does ATCC 19977

**Fig. 3.**
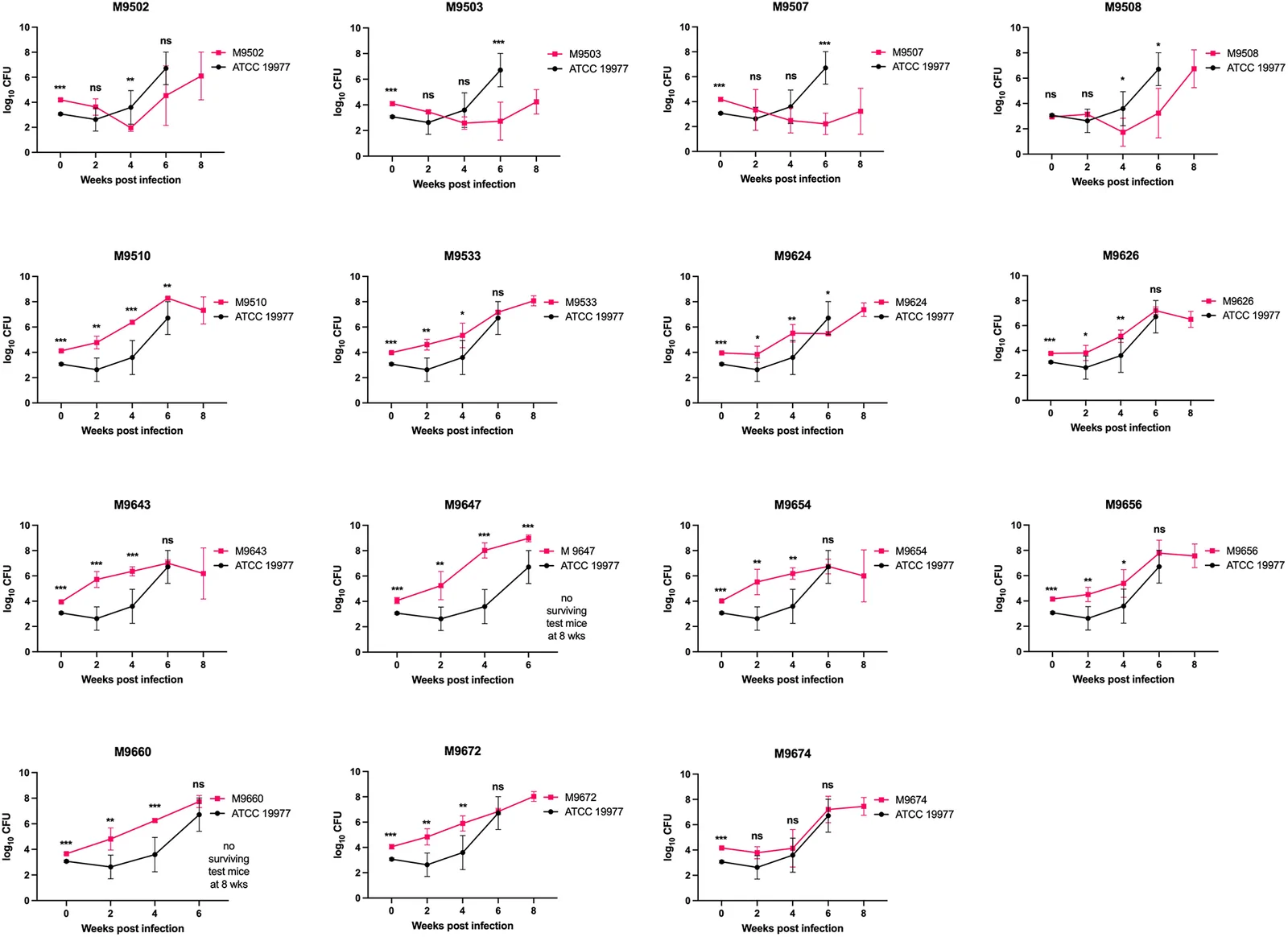
**Plots comparing mean burdens of** ***Mab*** **clinical isolates and ATCC 19977 in the lungs of C3HeB/FeJ mice.** Lung CFU burden of pulmonary-derived *Mab* isolates (pink) plotted individually against ATCC 19977 8+12 (black) after aerosol infection in mice. Each timepoint for each study represents five to eight animals. *P*-values by unpaired multiple *t*-testing with Welch correction, denoted by *P<0.05, **P<0.01, ***P<0.001 or ‘ns’ for nonsignificant.

All clinical *Mab* isolates established persistent infections in the mouse lung, although magnitude and rate of proliferation as determined by colony-forming unit (CFU) enumeration differed (Fig. 3; Fig. S5). Regardless of subspecies, pulmonary burdens for most isolates remained elevated during the early stages of infection and persisted through the 8-week endpoint, with all mice surviving to this final timepoint. M9647 and M9660 were exceptions, with all animals dying before the 8-week timepoint. Severity of structural lung damage also varied across isolates and generally correlated with the extent of *Mab* expansion (Table 2; Fig. S6).

**Table 2.** Lung histopathology findings at weeks 6–8 post infection for mice infected with clinical *Mab* isolates

Clinical isolate ID	Timepoints analyzed	Lung histopathology narrative and scores	Comparison to ATCC19977 lung pathology
M9502*	Week 8, 10	Neither lung at week 8 with visible structural lung disease (0, 0). At week 10, 1/2 samples with large AFB^+^ granuloma (0, 4).	Less structural lung disease at week 8 than for ATCC 19977, but comparable space-occupying lesions and structural damage at week 10 for M9502 as at week 6–7 for ATCC 19977.
M9503	Week 8, 10	No AFB organisms seen at any timepoint, and no structural lung disease identified (0, 0).	Less structural lung disease than for ATCC 19977.
M9507	Week 6, 8, 10	No AFB organisms or structural lung disease seen at any timepoint (0, 0).	Less structural lung disease than for ATCC 19977.
M9508	Week 6, 8, 10	Extensive AFB^+^ granulomatous disease at all timepoints (5, 5).	Substantially increased structural lung disease at weeks 8 and 10, with comparable disease at week 6 to that for ATCC 19977.
M9510*	Week 6, 8	At week 6, large-volume AFB^+^ granulomas seen in 2/2 samples (5, 5). Mice that survive to week 8 have some scarring, but no remaining AFB^+^ organisms seen (1, 1).	Substantially increased structural lung disease at week 6 than with ATCC 19977. Mice surviving to 8 weeks appear to have controlled disease but display evidence of prior infection.
M9533	Week 6, 8	Variably sized granulomatous disease in 1/2 samples at both timepoints (0, 4).	Comparable lung disease burden at week 6 of infection to that for ATCC 19977, with longer survival but less penetrance of lung disease at week 8.
M9624	Week 6, 8	1/2 samples at 6 weeks with single AFB^+^ lesion surrounding bronchus with invasion through mucosa. Other lung with no clear structural disease or AFB at 6 weeks (0, 3). No AFB or scarring at 8 weeks in either sample (0, 0).	Less structural damage and lower visible AFB burden at 6-week timepoint than for ATCC 19977. Mice surviving to 8 weeks appear to have controlled pulmonary disease.
M9626	Week 6, 8	Multiple large AFB^+^ granulomas in addition to bronchial AFB^+^ debris, perivascular invasion and free AFB (6, 6).	Substantially more structural damage to lungs at 6-week timepoint than for ATCC 19977 and with continued progression at 8-week timepoint.
M9643	Week 6, 8	For each timepoint, extensive AFB^+^ granulomas in 1/2 samples, but no AFB organisms or scarring/structural disruption seen in other sample (0, 5).	Comparable structural damage to lungs in some mice at 6-week timepoint, but with same pattern persisting at 8 weeks, showing less penetrance at that timepoint than for ATCC 19977.
M9647*	Week 6	Widespread, high-AFB-burden granulomas (variable sizes), with areas of hemorrhage, invasion into airways and substantial sclerosis in both week-6 samples (6, 6). No animals surviving at week 8 for analysis.	Substantially more structural damage to lungs at the 6-week timepoint than for ATCC 19977.
M9654	Week 6, 8	Moderate- to high-AFB-burden granulomas throughout lungs, with more structural damage seen at the 6-week timepoint (6, 6) than at the 8-week timepoint (5, 5).	Substantially more structural damage to lungs at the 6-week timepoint than for ATCC 19977. Mice that survive to 8 weeks begin to clear disease.
M9656	Week 6, 8	At week 6, 1/2 lungs with solitary small focus of AFB^+^ granulomatous change (0, 2). Lungs otherwise normal. No AFB seen at 8 weeks and only one small area of sclerosis in 1/2 samples (0, 1).	Less structural damage and lower visible AFB burden at 6 weeks compared to that for ATCC 19977, and disease resolving in animals surviving to week 8.
M9660	Week 6	Peribronchial and mucosal AFB invasion in one specimen, with paucibacillary granulomas in second specimen (3, 4).	Comparable to slightly increased structural lung disease at week 6 compared to that for ATCC 19977.
M9672	Week 6, 8	At week 6, 1/2 lungs with single moderately sized AFB^+^ granuloma in otherwise structurally normal lung. No evidence of structural disease at 8 weeks.	Comparable to less structural lung disease at week 6 than for ATCC 19977, and animals surviving to week 8 showed resolving disease.
M9674	Weeks 6, 8	1/2 specimens at 6 weeks and 2/2 specimens at 8 weeks with high-AFB-burden peribronchial and airway infiltration without organization into granulomas. Mats of AFB in airways.	Comparable AFB disease burden, with possibly less granuloma formation and more biofilm formation than for ATCC 19977 at 6 and 8 weeks.

Histopathological scores for each lung are reported in parentheses after their corresponding narrative description. Asterisks indicate the subspecies *massiliense* isolates. The remaining isolates are subspecies *abscessus*. AFB, acid-fast bacilli.

Isolates M9503 and M9507 produced low, but stable, lung burdens and showed no histopathologic evidence of lung injury. These characteristics suggest that such isolates can maintain chronic infections over extended periods and may represent useful models for studying chronic *Mab* pulmonary colonization. Several isolates produced more pronounced disease, with lung pathology by 6–8 weeks post-infection comparable to that seen with ATCC 19977. Subspecies *abscessus* isolates M9508, M9626, M9654 and M9674, along with subspecies *massiliense* isolates M9510 and M9647, induced more extensive structural lung damage and sustained higher burdens at terminal timepoints than did ATCC 19977. The lung burdens and pathology produced by these isolates more closely reflected those observed in patients with chronic *Mab* pulmonary disease (Table 2; Fig. S5).

#### Lung-derived isolates exhibit reduced systemic dissemination compared to ATCC 19977

**Fig. 4.**
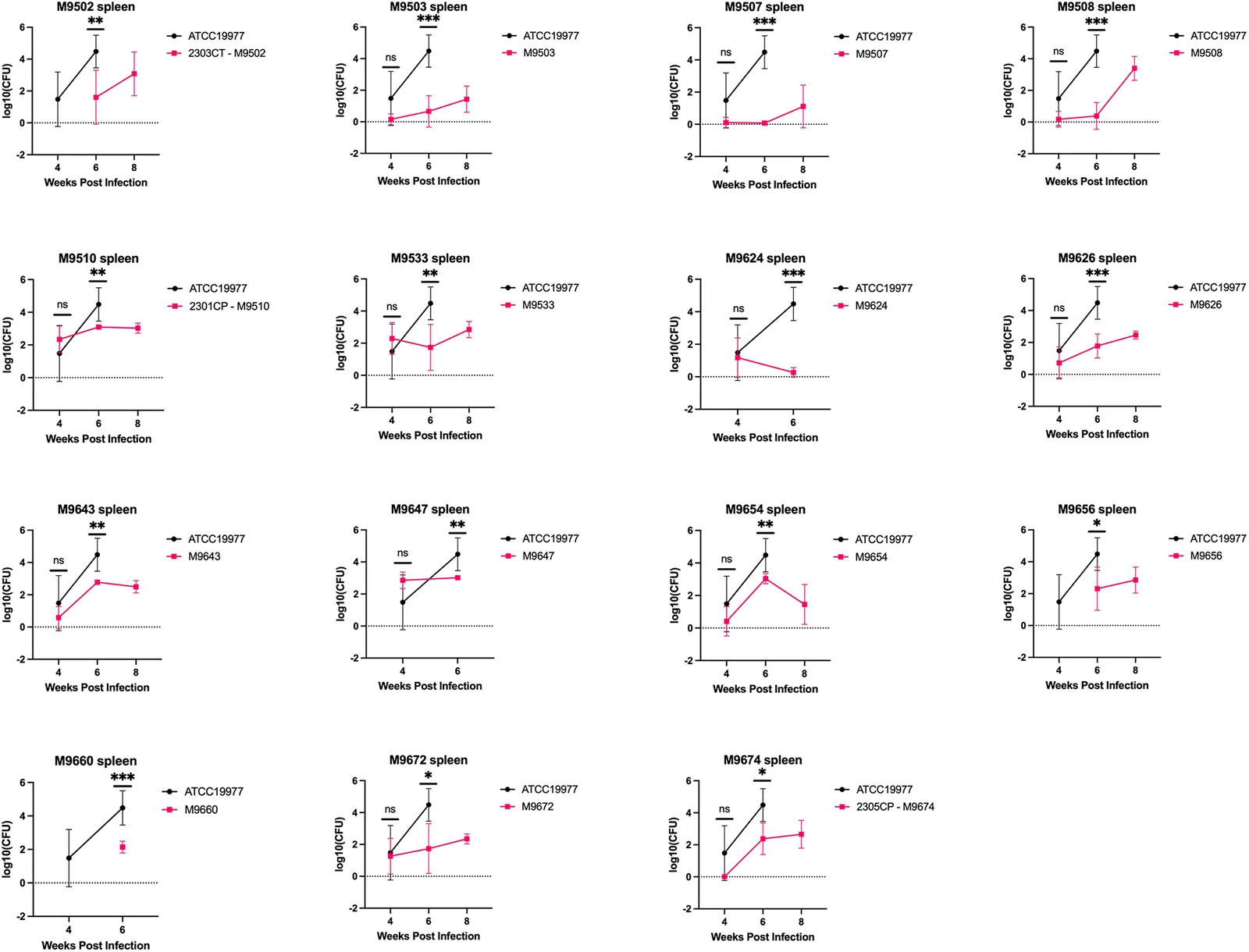
**Plots comparing mean burdens of *Mab* clinical isolates and ATCC 19977 in the spleen of C3HeB/FeJ mice.** Spleen CFU burden of pulmonary-derived *Mab* isolates (pink) plotted individually against ATCC 19977 8+12 (black) after aerosol infection in mice. Each timepoint for each study represents five to eight animals. *P*-values by unpaired multiple *t*-testing with Welch correction, denoted by *P<0.05, **P<0.01, ***P<0.001 or ‘ns’ for nonsignificant.

**Fig. 5.**
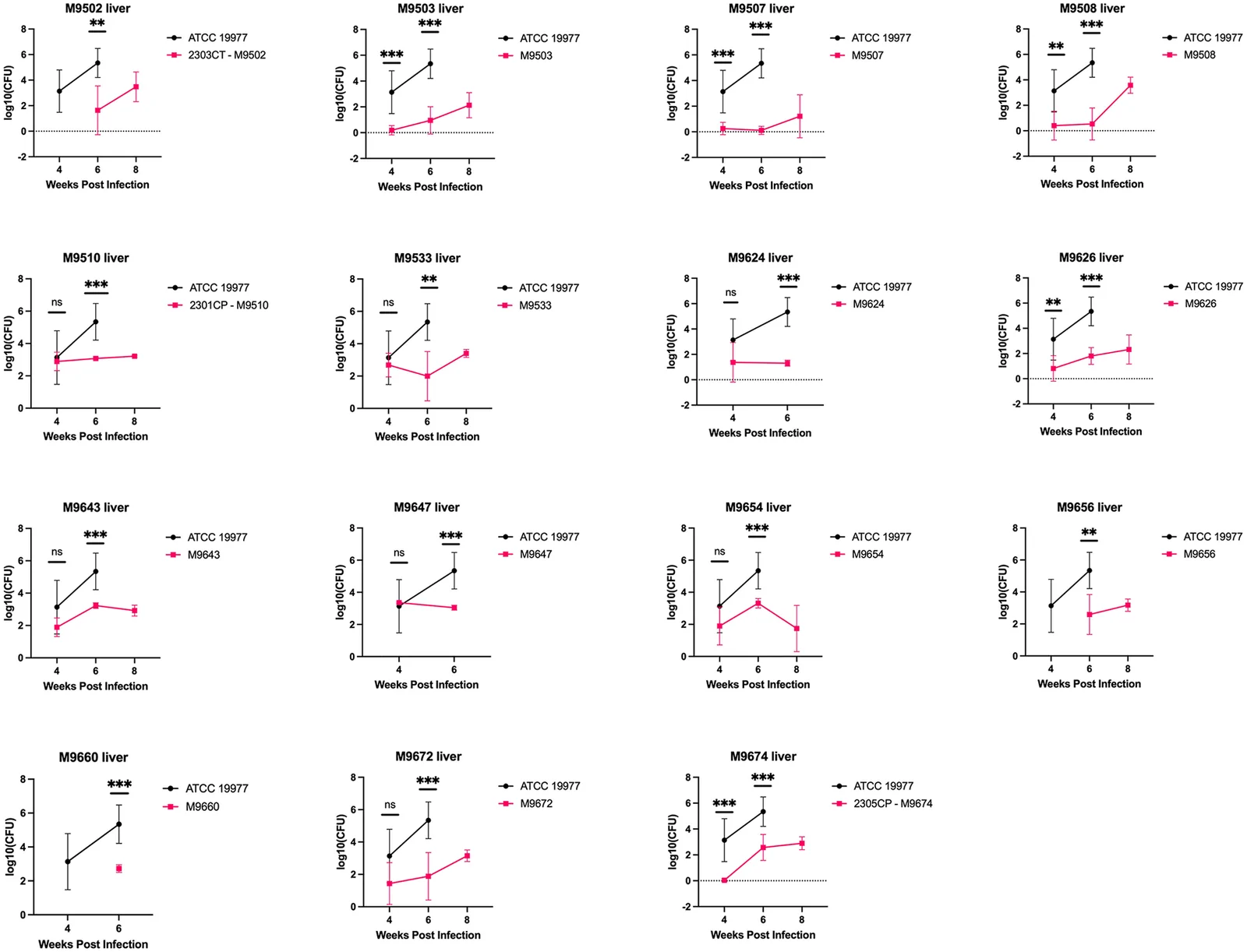
**Plots comparing mean burdens of** ***Mab*** **clinical isolates and ATCC 19977 in the liver of C3HeB/FeJ mice.** Liver CFU burden of pulmonary-derived *M. abscessus* isolates (pink) plotted individually against ATCC 19977 8+12 (black) after aerosol infection in mice. Each timepoint for each study represents five to eight animals. *P*-values by unpaired multiple *t*-testing with Welch correction, denoted by **P<0.01, ***P<0.001 or ‘ns’ for nonsignificant.

Infection with ATCC 19977 resulted in rapid onset of pulmonary lesions containing acid-fast bacilli (AFB) as early as 2 weeks post-infection. By 4 weeks post infection, these lesions showed evidence of tissue invasion within the lungs, accompanied by visible hepatic abnormalities, and by 6 weeks, the mice exhibited pronounced splenomegaly. CFU quantification confirmed systemic dissemination, with *Mab* burdens reaching ∼10^4^ CFU in spleen and 10^5^ CFU in liver at 6 weeks (Fig. 1B, Figs 4 and 5). In contrast, mice infected with clinical isolates displayed markedly reduced and delayed dissemination to both spleen and liver (Figs 4 and 5). Even among isolates associated with early mortality, such as M9647 and M9660, systemic bacterial loads were substantially lower, exceeding a 1 log_10_ reduction in spleen and more than a 2 log_10_ reduction in liver compared with ATCC 19977 at the same 6-week timepoint. These findings suggest that, unlike ATCC 19977, which readily disseminates, pulmonary-derived clinical isolates primarily cause localized disease confined to the lungs until late in infection. Consequently, mortality in these infections is likely to be driven by progressive pulmonary pathology rather than by systemic *Mab* spread.

## DISCUSSION

Reference strains that accurately represent contemporary *Mab* isolates from pulmonary infections are essential for advancing both fundamental and translational research. Most current *Mab* studies rely on the laboratory strain ATCC 19977, which was originally isolated from a knee abscess in 1950 (Moore and Frerichs, 1953). Although this strain has been instrumental in early *Mab* research owing to its availability, complete genome sequence, and well-characterized *in vitro* and *in vivo* properties, it does not represent the biological or clinical features of modern pulmonary isolates that now dominate patient infections.

Our findings demonstrate that ATCC 19977 differs markedly from contemporary lung-derived clinical isolates in virulence, disease localization and antibiotic susceptibility profiles. In mice, ATCC 19977 caused extensive early extrapulmonary dissemination, leading to high extrapulmonary burdens and significant mortality attributable to systemic disease. Within the lung, ATCC 19977 did not produce uniform pathology even at the later timepoints, further limiting opportunities to study pulmonary disease. These findings indicate that ATCC 19977 provides an unreliable model for subacute or chronic pulmonary infection, the clinical scenario most relevant to human disease. Consequently, preclinical studies that rely exclusively on this strain may require higher animal numbers to assess disease and produce results that may not accurately predict pathogenesis or treatment response in patients.

Over the past several decades, clinical and genomic studies have revealed extensive heterogeneity among *Mab* isolates, including differing morphotypes (smooth versus rough), virulence and subspecies (notably, *Mab* subspecies *abscessus* and *massiliense*) (Bohr et al., 2021; Gutiérrez et al., 2021; Yang et al., 2025). Each of these traits contributes to the wide range of disease phenotypes and treatment responses seen in patients. Given this diversity, a single reference strain is insufficient to capture the full biological and clinical spectrum of *Mab* pulmonary disease.

To address this gap, our study identified a panel of lung-derived, *Mab* isolates that more faithfully reproduce clinically relevant pulmonary disease characteristics. Based on comparative analyses of their *in vivo* lung growth profiles, associated histopathologic changes, dissemination patterns, colony morphotypes and antibiotic susceptibility profiles, five *Mab* isolates emerged as compelling candidates to serve as new reference strains (Table 3). We propose that *Mab* subspecies *abscessus* strains M9508, M9626 and M9654, along with *Mab* subspecies *massiliense* strains M9510 and M9647, together constitute a more representative and tractable reference set for modeling *Mab* pulmonary infection (Table 3). This set encompasses both major subspecies and includes smooth, rough and mixed morphotypes. Importantly, each demonstrated robust lung colonization and high pulmonary disease penetrance in mice, confirmed by both histopathology and lung CFU enumeration, while exhibiting minimal and delayed systemic dissemination compared to ATCC 19977. They were, therefore, selected as representative of contemporary *Mab* strains, the biological and pathogenic characteristics of which more closely reflect those encountered in current patient populations. Not all clinical scenarios can be foreseen, but these strains may be matched to modeling specific scenarios:

**Table 3.** Summary of recommended strains for high-penetrance modeling of *Mab* subspecies *abscessus* and *massiliense* pulmonary disease and primary characteristics

Isolate ID	Subspecies	Composite morphotype	Lung CFU burden compared to that for ATCC 19977	Disease penetrance by 8 weeks	Systemic dissemination	Median CLR MIC (μg/ml)
M9508	*abscessus*	Rough	Higher burden, delayed onset	100%	Some, late in course	24
M9626	*abscessus*	Mixed	Higher burden	100%	Some, late in course	24
M9654	*abscessus*	Smooth	Higher burden	100%	Some, late in course	20
M9510	*massiliense*	Smooth	Higher burden	100%	Some, late in course	4
M9647	*massiliense*	Rough	Higher burden	100%	Some, late in course	10

‘Composite morphotype’ refers to combined behavior in liquid and on solid media (Table 1). CFU, colony-forming unit; CLR, clarithromycin; MIC, minimum inhibitory concentration.

### *Mab* subspecies *abscessus* M9508 (rough morphotype on agar; planktonic in broth)

M9508 produced progressive lung disease characterized by structural tissue damage evident by 8 weeks post-infection, with animals surviving through 10 weeks and showing late-stage systemic dissemination. These features make M9508 well suited for modeling chronic *Mab* pulmonary disease with rough-strain conversion and secondary dissemination, as seen in older patients with cystic fibrosis and advanced infection.

### *Mab* subspecies *abscessus* M9626 (mixed morphotype on agar; planktonic and biofilm growth in broth)

M9626 caused more rapid onset of pulmonary disease than did M9508 but exhibited limited extrapulmonary spread. Its faster disease progression and primarily lung-confined pathology make it a valuable model for studying acute or rapidly progressive infections, such as those occurring in transplant recipients, and for evaluating inhaled therapeutic interventions with minimal systemic dissemination.

### *Mab* subspecies *abscessus* M9654 (smooth morphotype on agar; planktonic in broth)

The disease course of M9654 was similar to that of M9626, but it had slightly lower macrolide MICs. This would make M9654 particularly useful for testing macrolide-based combination therapies or regimens targeting early or less disseminated forms of *Mab* lung disease.

### *Mab* subspecies *massiliense* M9510 (smooth morphotype on agar; planktonic in broth)

M9510 exhibited low macrolide MICs associated with an inactive *erm(41)* gene. It produced high pulmonary bacterial burdens but minimal dissemination, making it an excellent model for localized *M. massiliense* infections dominated by smooth morphotypes.

### *Mab* subspecies *massiliense* M9647 (rough morphotype on agar; planktonic and biofilm growth in broth)

Despite a non-functional *erm(41)* gene, the macrolide MICs were slightly higher than those for M9510, possibly because of its extensive biofilm formation. M9647 maintained high pulmonary bacterial loads with limited systemic spread but exhibited rough colony morphology associated with enhanced tissue invasion, inflammation and early mortality. M9647 modeled rough morphotype-driven *M. massiliense* disease that progresses more rapidly but remains primarily pulmonary.

Both *massiliense* strains (M9510 and M9647) demonstrated faster pulmonary disease progression with less systemic dissemination than the *abscessus* isolates, making them suitable for evaluating inhaled and systemic (oral or injectable) therapeutic regimens. These isolates are genetically diverse, consistent with the large and open pan genome of *Mab*. Although this study included only isolates from patients in North America, we found that the *massiliense* isolates clustered with *Mab* genomes from multiple sites in Southeast Asia, suggesting that they may still be of clinical utility for modeling disease in global populations. However, as the field encounters new challenges, we anticipate that more models will need to be developed.

Using these strains in preclinical research offers several advantages. High and consistent lung disease penetrance enables efficient experimental design, reducing the number of animals and resources needed to achieve meaningful results. Moreover, limited extrapulmonary spread minimizes confounding mortality from extrapulmonary disease, thereby improving the interpretability of survival and treatment efficacy data in therapeutic studies, particularly those evaluating inhaled treatments. Although inclusion of all five strains is feasible for *in vitro* studies, *in vivo* experiments require prioritization. We recommend M9508, M9626 and M9510, which collectively represent both subspecies, colony morphotypes and a range of lung burdens. If limited to one strain, M9626 is preferred for the versatility of its mixed morphotype.

The current immunosuppression regimen employs higher doses of dexamethasone than those used in our initial model to support sustained growth of contemporary lung-derived *Mab* isolates. In the original model, once-daily administration of 5 mg/kg dexamethasone was sufficient to support growth of ATCC 19977 in C3HeB/FeJ mouse lungs for ∼3–4 weeks, and we have applied this regimen in multiple prior studies. Although absolute lung CFU values have varied between experiments, the overall trend, a gradual increase in bacterial burden over time, has been consistent and reproducible. In the present study, we observed pulmonary engraftment of 3.0–4.2 log_10_(CFU) 1 day post infection, consistent with the ranges observed in our prior studies using lower dexamethasone doses. However, as illustrated by the ATCC 19977 data in this study, one must be careful to avoid direct comparison of infection dynamics between experiments using different immunosuppressive regimens. Notably, several clinical isolates also exhibit distinct growth patterns in mouse lungs when different dexamethasone regimens are used, highlighting the importance of tailoring immunosuppression to the strain under investigation.

A major challenge in conducting any animal experiment on the scale of the present study is controlling for biological variability and batch effect. It is beyond the scope of an academic laboratory to handle nearly a thousand mice simultaneously for daily injections, so temporal separation of experiments remains a necessary source of experimental variability. In our experience, it is also difficult to source even 100–200 C3HeB/FeJ mice from a single breeding facility at a time, and we had several orders filled partially from two different breeding facilities serving the same supplier, which may be an under-recognized source of variability for large experiments. The ethical principle of minimizing unnecessary animal use adds an additional layer of complexity when designing controls for long-running animal experiments, such as the ATCC 19977 comparator arm in this study. This work was conducted over 4 years, so although all animals were ordered from the same supplier, their arrivals at different times of the year and from different breeding facilities at slightly different ages is expected to impact study results in non-controllable ways. For this reason, although we have attempted to control for cohort and individual biological variability through experimental design, the gold standard validation for this type of animal model work remains validation by multiple independent laboratories. To this end, we have committed to making the *Mab* strains used in this study available to independent academic groups.

Although our present work focused on establishing these strains within a standardized murine infection model to directly compare their pathogenic behaviors, this reference set can be adapted for use in diverse experimental contexts. For example, they could be integrated into immunocompetent or immunocompromised mouse models, aerosol infection systems, or studies exploring host immune responses and drug pharmacodynamics. Although we find the most robust, humanoid pathology with immunosuppressed C3HeB/FeJ mice, other models may be better suited to particular questions or laboratory settings. We anticipate that these *Mab* strains would be adaptable with additional validation. Ultimately, adopting this broader, biologically relevant panel of *Mab* reference strains will enhance the translational relevance of preclinical studies and accelerate progress toward effective therapies for this increasingly prevalent and difficult-to-treat respiratory pathogen.

## MATERIALS AND METHODS

### Ethics

Mouse handling and procedures were undertaken in adherence with the national and the Animal Care and Use guidelines approved by the Johns Hopkins University Animal Care and Use Committee (protocol number MO23M123).

### Bacterial strains, culture media, drugs and growth conditions

*Mycobacterium abscessus* (*Mab*) strain ATCC 19977 (Moore and Frerichs, 1953), was procured in 2014 from American Type Culture Collection (Manassas, VA, USA) and authenticated as belonging to the subspecies *abscessus* (Story-Roller et al., 2021). *Mab* isolates M9502, M9503, M9507, M9508, M9510 and M9533 were isolated from lung expectorates obtained from patients with bronchiectasis and cystic fibrosis at the Johns Hopkins Hospital (JHH; Baltimore, MD, USA) between 2004 and 2020 and archived by the Johns Hopkins Clinical Mycobacteriology Laboratory (JHCML). Similarly, *Mab* isolates M9624, M9626, M9643, M9647, M9654, M9656, M9660, M9672 and M9674 were obtained from lung samples from patients with and without cystic fibrosis at National Jewish Health (NJH; Denver, CO, USA) between 2019 and 2021 and archived by the Clinical Mycobacteriology Laboratory. For isolates originating from JHH, their genomes were sequenced, and these data were used to determine their subspecies classification as described (Story-Roller et al., 2021). Similarly, the NJH Clinical Mycobacteriology Laboratory performed whole-genome sequencing and subspecies classification for isolates originating at that institution.

All *Mab* isolates were inoculated from early-passage frozen stocks (P1–P3 from laboratory acquisition) and cultured in Middlebrook 7H9 broth (Difco, 271310) supplemented with 0.5% glycerol, 10% albumin–dextrose–sodium chloride (ADS) enrichment and 0.05% Tween-80 at 37°C in an orbital shaker at 220 rpm (Larsen, 2000) except for Tween-free experiments.

Powdered form of drugs were purchased or donated from commercial vendors as follows: amikacin (Sigma-Aldrich, A3650), amoxicillin (Sigma-Aldrich, A8523), azithromycin (Sigma-Aldrich, 75199), bedaquiline (MedChemExpress, HY-14881AR), cefdinir (VWR, TCC3111), cefditoren (Sigma-Aldrich, SML3624), cefoxitin (Sigma-Aldrich, C4786), ceftazidime (Sigma-Aldrich, C3809), cefuroxime (Sigma-Aldrich, C4417) clarithromycin (Sigma-Aldrich, C9742), clofazimine (Sigma-Aldrich, C8895), dicloxacillin (Sigma-Aldrich, D9016), doripenem (Sigma-Aldrich, SML1220), faropenem (Octagon Chemicals), imipenem (Sigma-Aldrich, I0160), linezolid (Octagon Chemicals), metronidazole (Sigma-Aldrich, M3761), moxifloxacin (Sigma-Aldrich, PHR1542), MRX-6038 (MicuRx Pharmaceuticals), omadacycline (Paratek Pharmaceuticals), oxacillin (Sigma-Aldrich, 28221), penicillin V (Sigma-Aldrich, PHR2644), rifabutin (Sigma-Aldrich, R3530) and tebipenem (Octagon Chemicals).

### Smooth and rough colony morphotyping

*Mab* isolates were cultured simultaneously under standardized liquid and solid growth conditions to ensure consistent morphological assessment. To assess phenotype in standard liquid culture, isolates were inoculated into Middlebrook 7H9 broth supplemented with 0.5% glycerol, 10% ADS and 0.05% Tween-80 to minimize clumping. Cultures were incubated at 37°C under aerobic, shaking conditions (220 rpm) in test tubes measuring 1–1.5 cm in internal diameter. Tubes composed of different materials (borosilicate glass, polystyrene, polypropylene or resin) were used to evaluate planktonic or clumping growth behavior and formation of meniscal biofilms with or without wall-climbing behavior. To assess liquid culture phenotype in the absence of detergent, log-phase *Mab* cultures were prepared in Tween-containing media, then a 1 μl loop was used to inoculate glass culture tubes containing 2 ml Middlebrook 7H9 broth supplemented with 0.5% glycerol and 10% ADS and incubated as above.

For smooth versus rough colony phenotyping, *Mab* cultures from mid-log phase in planktonic form were streaked onto Middlebrook 7H10 agar supplemented with 0.5% glycerol and 10% ADS. Plates were incubated at 37°C for 3–5 days, or until discrete, well-isolated colonies were visible. Colony morphology was then assessed both macroscopically and microscopically. Visual inspection was performed under front- and back-lighting conditions, and images were recorded using an imager (Scan 300, Interscience). Morphotypes on solid media were categorized as follows. Smooth morphotype: colonies appeared glabrous, dome-shaped and circular, with a smooth, moist or oily surface sheen. Rough morphotype: colonies exhibited an irregular circumference, flat or flaky texture and a dry, matte appearance. All phenotyping assessments were performed on at least two independent biological replicates to ensure reproducibility. Composite morphotype was determined to be the overall behavior between solid media and liquid media across all tube materials. The composite morphotype is slightly different from the solid media morphotype under certain conditions to account for mixed or non-classical behavior patterns. Specifically, composite morphotype ‘rough’ included strains forming colonies with mixed characteristics on solid media, but noted to have more aggressive aggregation and biofilm formation in liquid media. When solid media morphotype was mixed and the dominant liquid media growth habits ranged from pure planktonic to minimal aggregate or biofilm formation, the composite morphotype for the strain was termed as ‘mixed’.

### MIC determinations

The MIC of each drug against individual *Mab* clinical isolates was determined using the standard broth microdilution method (Cynamon et al., 1998; Tilton et al., 1973), based on CLSI guidelines specific to *Mab* (CLSI, 2023). Briefly, powdered drug stocks were reconstituted in dimethyl sulfoxide, and twofold serial dilutions were prepared in Middlebrook 7H9 broth supplemented with 0.5% glycerol and 10% ADS to yield final drug concentrations ranging from 128 μg/ml to 0.125 μg/ml for amikacin, amoxicillin, azithromycin, cefdinir, cefditoren, cefoxitin, ceftazidime, cefuroxime, clarithromycin, dicloxacillin, doripenem, faropenem, imipenem, linezolid, metronidazole, moxifloxacin, oxacillin, penicillin V, rifabutin and tebipenem, and from 16 μg/ml to 0.016 μg/ml for bedaquiline, clofazimine, MRX-6038 and omadacycline. Each well of a round-bottomed 96-well microtiter plate (final volume, 200 μl) was inoculated with ∼10^5^ CFU of *Mab* from a log-phase culture. Drug-free *Mab* cultures and broth-only wells were included on each plate as positive and negative controls, respectively. Plates were incubated at 30°C for 72 h according to CLSI guidelines, then assessed using a Sensititre Manual Viewbox. The MIC was defined as the lowest drug concentration that completely inhibited visible *Mab* growth. All MIC determinations were performed with technical duplicates for each biological replicate. Assays were performed in a minimum of two biological duplicates except for faropenem, tebipenem, metronidazole, penicillin V, cefditoren, MRX-6038 and omadacycline, for which supplies were limiting. Additional biological replicates were performed if there was a greater than two-well discrepancy until a median MIC could be established and reproduced. Median MIC values calculated from the replicates are reported.

### Mouse infections and *Mab* burden determination

Female C3HeB/FeJ (Kramnik) mice (4–6 weeks old at the time of shipment from The Jackson Laboratory; strain #000658) were used for all experiments as described (Maggioncalda et al., 2020). Each infection was performed at a different time with separate mouse cohorts over a 4-year period. Upon arrival, animals were housed under biosafety level 2 (BSL-2) conditions and acclimated for 10–14 days before experimentation. To induce immunosuppression, dexamethasone was administered once daily, 7 days per week. Dexamethasone powder (Sigma-Aldrich, D1756) was weighed for daily dosing and stored at −20°C in 25 ml polypropylene tubes. Immediately before administration, the powder was reconstituted in 1× PBS (pH 7.4) to the desired final concentration and vortexed at high speed for 2 min, yielding a cloudy white homogeneous suspension. 200 μl of this suspension was administered subcutaneously into the hind dorsal flank using a 27-gauge needle (Becton Dickinson, 305620). On day 7 after treatment initiation, mice were infected with *Mab* via aerosol using an Inhalation Exposure System (Model A4212, Glas-Col, Terre Haute, IN, USA) equipped with a Collison-type nebulizer. A total of 11 ml of *Mab* culture grown in Middlebrook 7H9 broth to log phase and diluted to optical density at 600 nm (OD_600_)=0.1 was nebulized following a standard cycle: 15 min preheating, 30 min nebulization, 30 min cloud decay and 15 min UV surface decontamination. After exposure, animals were returned to individual cages and maintained under BSL-2 housing.

To determine lung implantation, five mice were euthanized 1 day post-infection (week 0), and their lungs were collected aseptically. Additional groups (five mice at week 2 and eight per subsequent timepoint) were sacrificed for histopathological analysis and determination of infectious burden. Lungs, livers and spleens were placed in sterile tubes containing 2 mm glass beads and phosphate-buffered saline (PBS; pH 7.4), then homogenized using a Minilys bead beater (Bertin Instruments) for 30 s at 4000 rpm. Homogenates were tenfold serially diluted in PBS, and 100 μl aliquots were plated on Middlebrook 7H11 agar supplemented with 50 μg/ml carbenicillin (Research Products International, C46000) and 50 μg/ml cycloheximide (Sigma-Aldrich, C7698). Plates were incubated for 7 days at 37°C, and colonies were enumerated. CFU counts were adjusted for dilution, averaged among technical replicates, and expressed as log_10_(CFU) for plotting and statistical analysis.

### Histopathology

Two whole livers, two whole spleens and one lung from each of two mice were collected per timepoint for histopathology. Organs were fixed in 10% neutral buffered formalin for at least 24 h, then either stored in PBS at 4°C or submitted directly for processing. Tissues were paraffin embedded and sectioned at 4–5 μm, and serial slices were stained with Hematoxylin and Eosin, Masson trichrome and Ziehl–Neelsen (AFB) stains at the JHH Oncology Tissue Services Core. Slides were digitized with unique identifiers using a high-resolution scanner and analyzed in a masked manner. Histopathological scoring based on these narrative reports was performed with 1 point given for each feature to a maximum of 6 points: presence of AFB organisms in tissue; multiple foci of infection; microscopic distortion of normal organ structure related to AFB organisms; gross distortion of normal organ structure related to AFB organisms; presence of abnormal collagen deposition or mature granulomas; and presence of gross organ pathology of indeterminate cause. Representative images and corresponding descriptions were subsequently reassociated with their respective infection groups for data interpretation.

### Phylogenetic analysis

Genomes of strains isolated at JHH were obtained as previously described (Story-Roller et al., 2021). Paired-end Illumina reads for strains isolated at NJH were provided by the laboratory of Charles Daley and *de novo* assembled using the Geneious assembler, then mapped to the ATCC 19977 NCBI reference sequence NC_010397 using Geneious mapping. Alignments for all strains were performed using Progressive Mauve (Darling et al., 2010). A phylogenetic tree was constructed in the Geneious tree builder using Tamura-Nei distance modeling with neighbor joining and 100 iterations of bootstrap resampling. *Mycobacterium avium hominissuis* type strain OCU889s was used to anchor the tree. Additional outgroups *massiliense* JCM 15300 type strain (Nakanaga et al., 2014) and international clinical pulmonary *abscessus* isolates N40 and M24 (Choo et al., 2014; Jin et al., 2022; Ngeow et al., 2012) were used to contextualize global generalizability.

### Data analysis

Mean log_10_(CFU) counts for each biological replicate were analyzed in Prism (version 10, GraphPad Software). Log_10_(CFU) data for clinical *Mab* isolate experiments were compared to the ATCC 19977 8+12 isolate experiment using by unpaired multiple *t*-testing with Welch correction at each timepoint. *P*<0.05 was considered statistically significant.

## Supplementary Material

10.1242/dmm.052759_sup1 Supplementary information
