# Supplementary material for: Expanding the scope of Mycobacterium abscessus reference strains to improve pulmonary disease modeling
Source: Dis Model Mech. 2026 Sep 1;19(8):dmm052759. doi: 10.1242/dmm.052759 (PMC13580562; doi:10.1242/dmm.052759)
Supplement: Supplementary file 1 [file dmm-19-052759-s1.pdf]

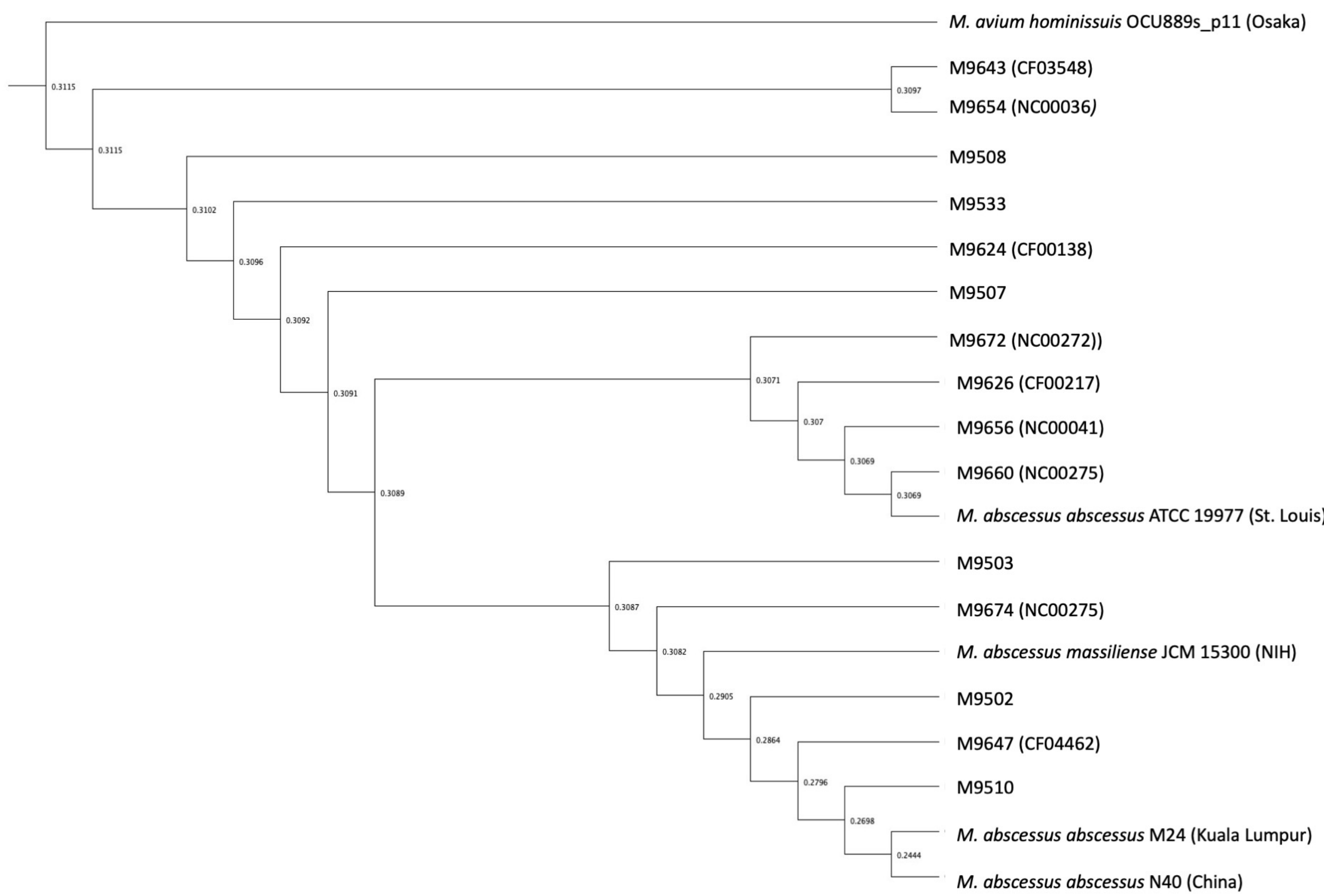

Fig. S1. Phylogenetic tree of *M. abscessus* strains in this study based on whole genome alignment. Outgroups including *M. avium hominissuis* OCU889s, *M. abscessus* subspecies *massiliense* JCM 15300, and international clinical pulmonary *M. abscessus* subspecies *abscessus* isolates N40 and M24 are included for context. Branches are transformed proportionally without label alignment for ease of viewing tight clusters, with node heights indicated at each node.

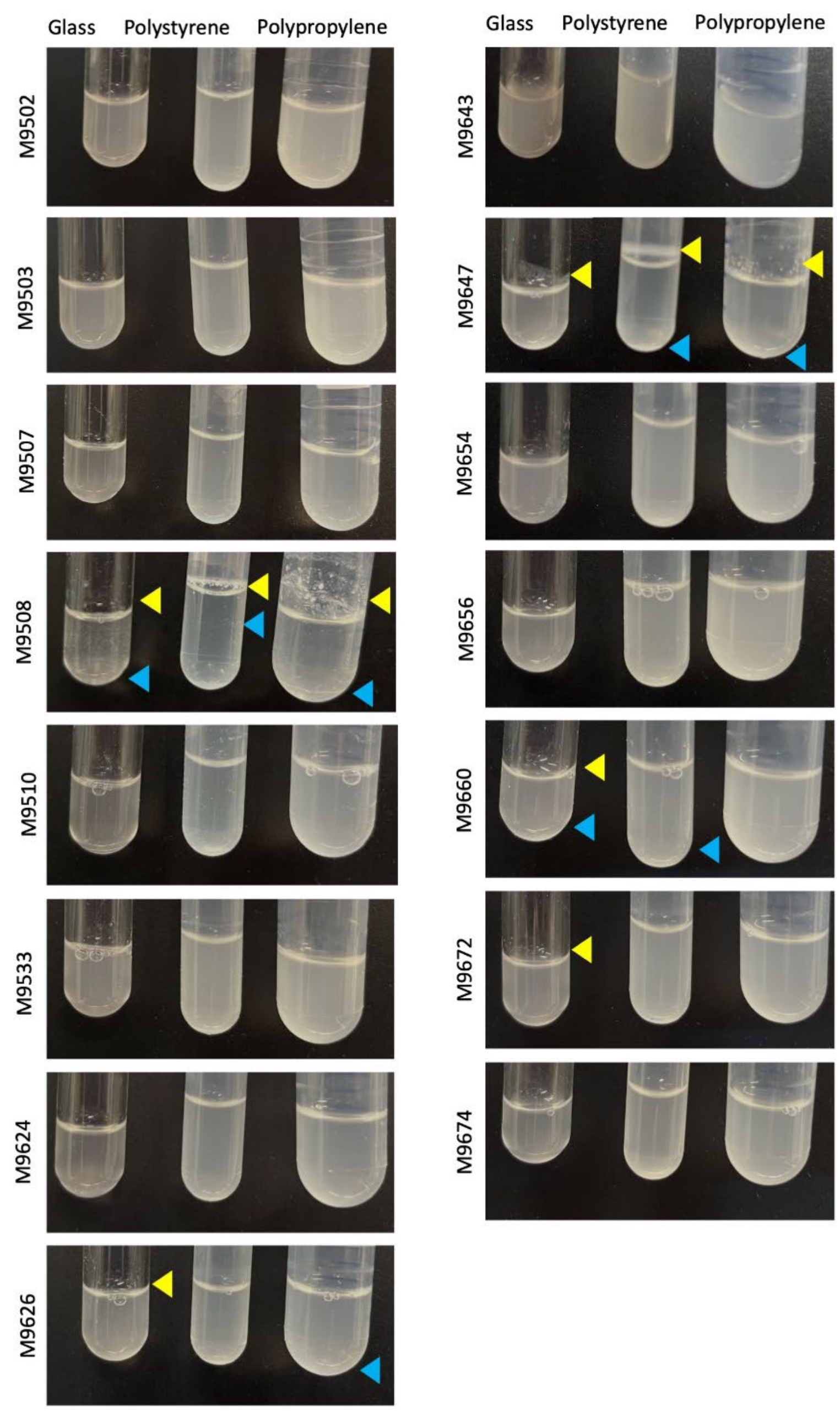

Fig. S2. **Liquid growth characteristics of pulmonary derived *M. abscessus* isolates grown in Middlebrok 7H9 media supplemented with glycerol, ADC, and Tween80 in tubes made with material of different porosity.** Yellow arrowheads indicate meniscal biofilm or liminal colony formation. Blue arrowheads indicate non-planktonic aggregates within the liquid media.

Disease Models & Mechanisms • Supplementary information

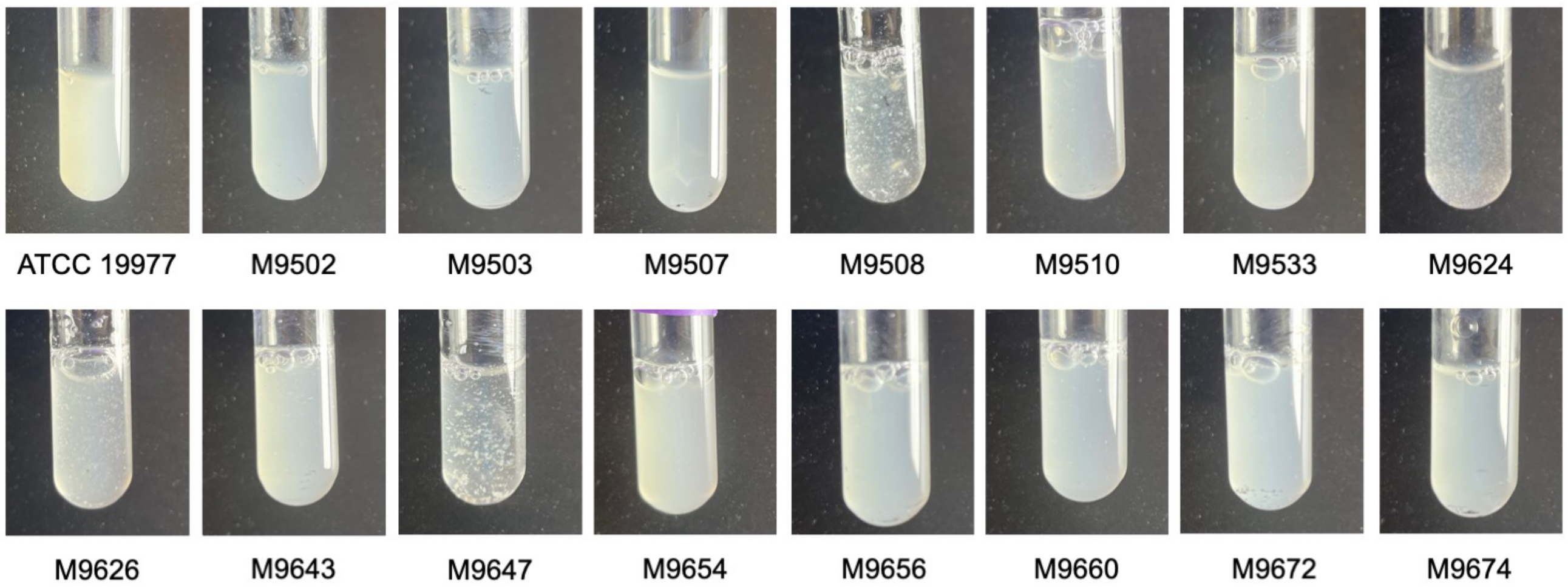

Fig. S3. **Liquid culture phenotype of *M. abscessus* in the absence of detergent.** Liquid culture phenotypes of *M. abscessus* grown in Tween-free media in glass vials display a range of phenotypes from pure planktonic to oily meniscal films to flocculent aggregate. None display wall-climbing biofilm phenotypes in glass vials.

Disease Models & Mechanisms • Supplementary information

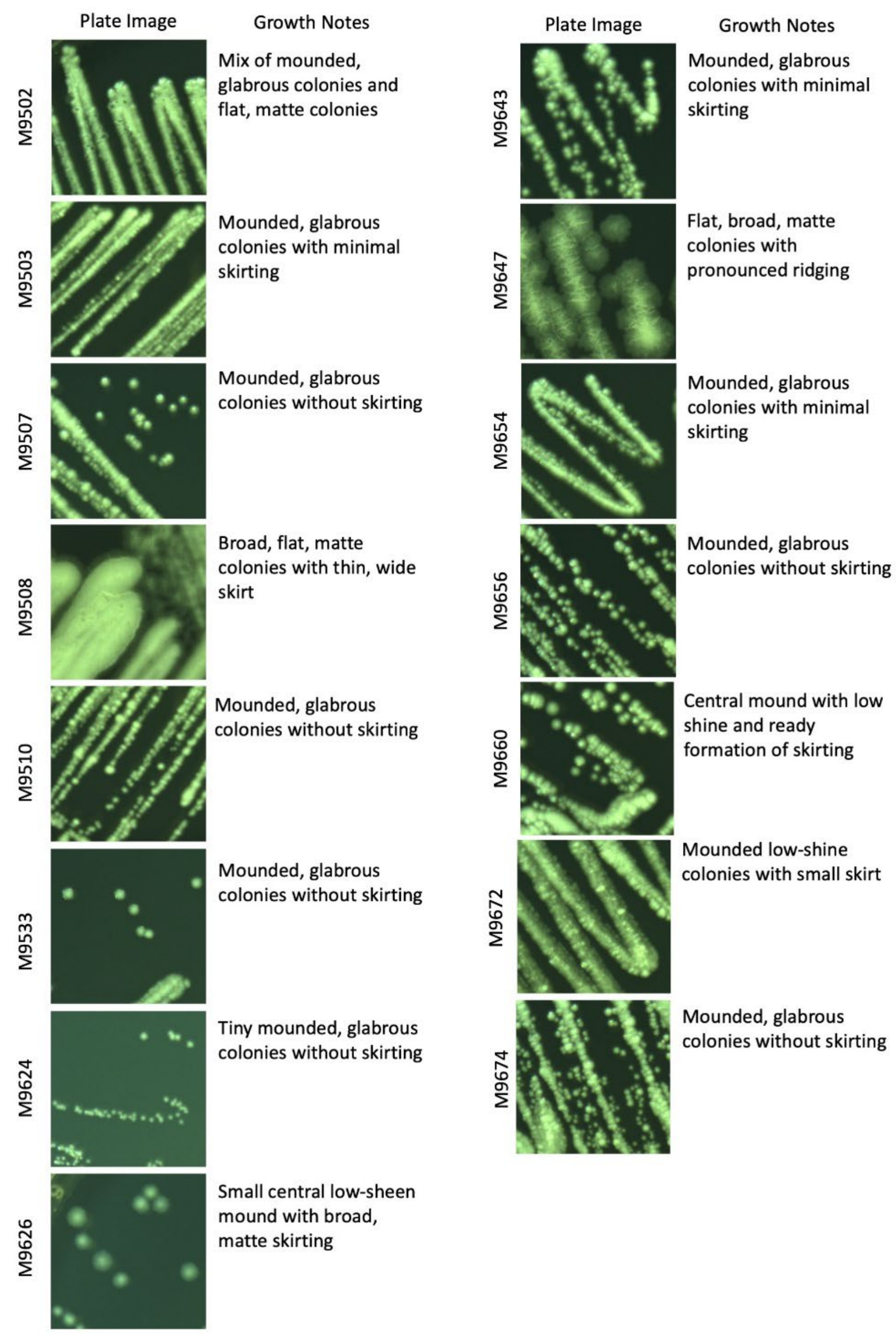

Fig. S4. **Colony morphologies of pulmonary-derived *M. abscessus* isolates grown on Middlebrook 7H10 plates supplemented with glycerol and ADC.**

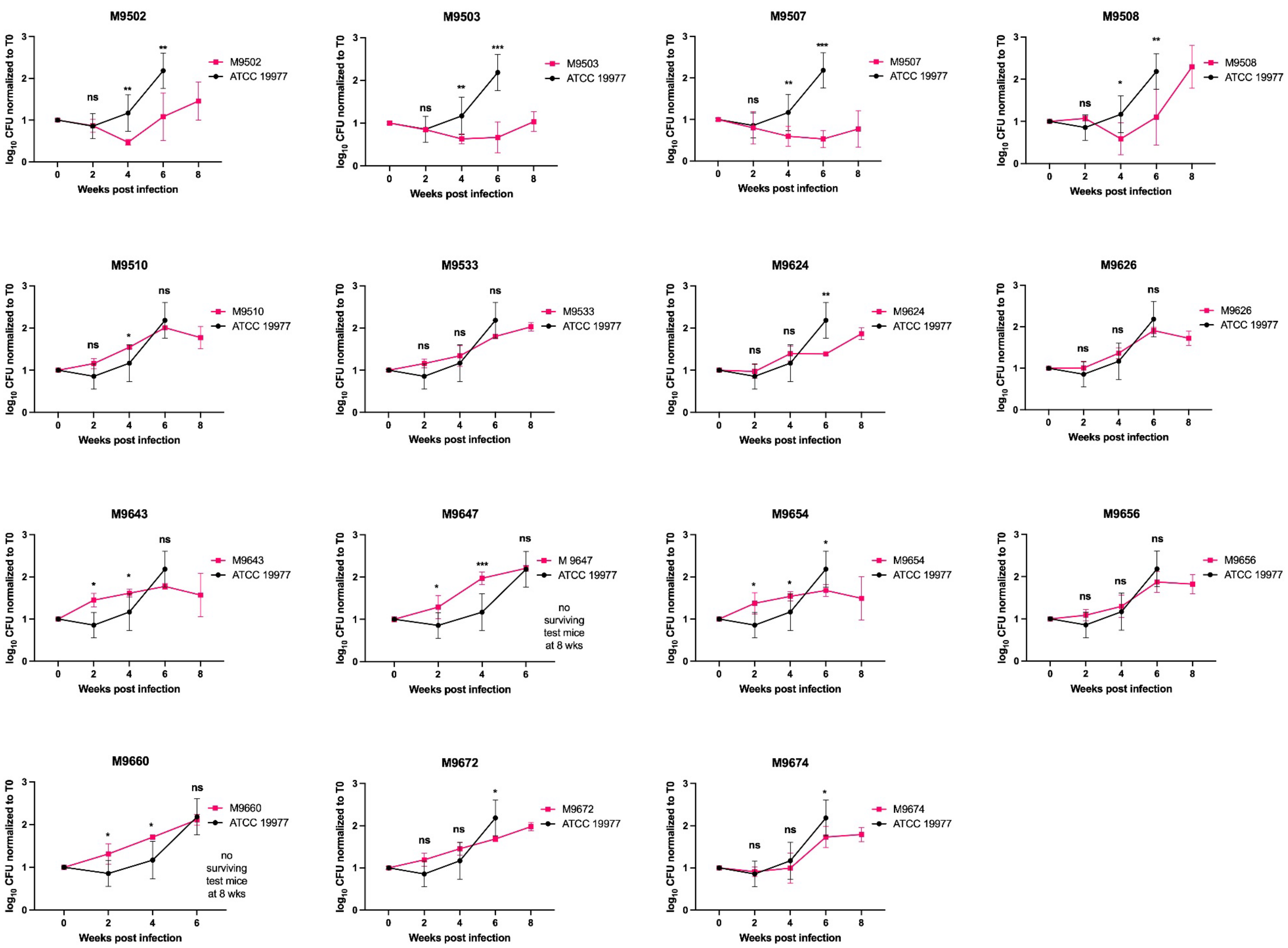

Fig. S5. Normalized lung CFU burden of pulmonary-derived *M. abscessus* isolates (pink) plotted individually against ATCC 19977 8+12 (black) after aerosol infection in mice. Each timepoint for each study represents 5-8 animals. All values were normalized to the mean log10(CFU) value for that study at T=0. Statistical significance is reported as p-value with *= <0.05, **=<0.01, ***=<0.001 or ns denoting nonsignificant based on multiple unpaired t-testing with Welch correction.

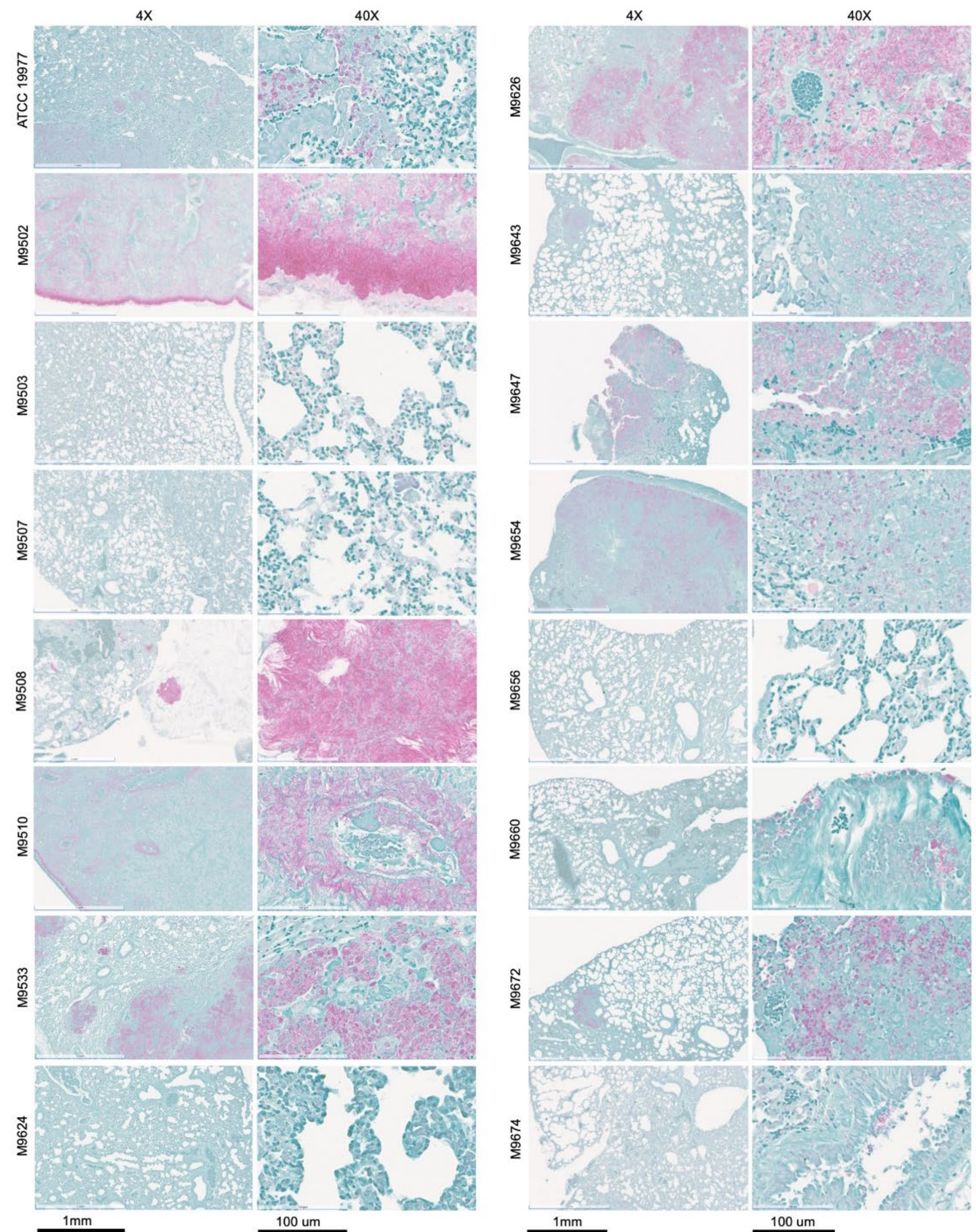

Fig. S6. Lung histopathology for ATCC 19977 and clinical pulmonary *M. abscessus* isolates at study endpoint. Representative AFB-stained lung sections are shown at 4x and at 40x magnification for the same section. In this stain, bright magenta bacilli growing independently, in clumps, within macrophages, or in cords indicate *M. abscessus* presence. Host cells are stained light blue with faded pink nuclei.

Table S1. Median MIC values for antibiotics for ATCC 19977 and clinical *Mab* isolates. Abbreviations: CLR clarithromycin; AZM azithromycin; IMI imipenem; DOR doripenem; FRP faropenem; TBP tebipenem; AMK amikacin; CFZ clofazimine; MET metronidazole; LZD linezolid; MOX moxifloxacin; RFB rifabutin; OMC omadacycline; BDQ bedaquiline; PVK penicillin V; AMX amoxacillin; OXA oxacillin; CXM cefuroxime; FOX cefoxitin; CPD cefpodoxime; CDR cefdinir; CAZ ceftazidime.

Drug Class	Macrolide	Macrolide	Carbapenem	Carbapenem	Carbapenem	Carbapenem	Aminoglycoside	Phenazine	Nitroimidazole	Oxazolidinone	Fluoroquinolone	rifamycin	tetracycline	Benzoxaborole	Diarylquinoline	Penicillin, 1st	Aminopenicillin	Penicillin, 2nd	Penicillin, 2nd	Cephalosporin, 2nd	Cephamycin	Cephalosporin, 3rd	Cephalosporin, 3rd	Cephalosporin, 3rd
	**CLR**	**AZM**	**IMI**	**DOR**	**FRP**	**TBP**	**AMK**	**CFZ**	**MET**	**LZD**	**MOX**	**RFB**	**OMC**	**MRX-6038**	**BDQ**	**PVK**	**AMX**	**OXA**	**DCX**	**CXM**	**FOX**	**CPD**	**CDR**	**CAZ**
**Isolate**																								
**ATCC 19977**	24	16	20	24	128	128	32	4	>128	128	16	32	0.5	0.25	0.095	>128	>128	>128	>128	>128	64	128	128	>128
**M9502**	3	24	16	40	128	128	48	4	>128	96	20	24	0.5	0.13	0.095	>128	>128	>128	>128	>128	32	>128	48	>128
**M9503**	8	32	32	40	128	128	24	8	>128	96	24	24	0.5	0.25	0.155	>128	>128	>128	>128	128	24	128	64	>128
**M9507**	80	>128	32	96	>128	128	8	4	>128	>128	40	12	0.5	0.13	0.07	>128	>128	>128	>128	>128	48	>128	>128	>128
**M9508**	24	128	12	32	128	128	16	4	>128	16	16	36	1	0.25	0.28	>128	>128	>128	>128	>128	24	128	96	>128
**M9510**	4	96	32	96	>128	128	16	2	>128	96	80	12	1	0.5	0.033	>128	>128	>128	>128	>128	80	128	96	>128
**M9533**	40	128	8	24	128	128	48	8	>128	96	12	24	0.5	0.25	0.095	>128	>128	>128	>128	>128	48	128	96	>128
**M9624**	>128	>128	>128	>128	>128	128	16	4	>128	80	96	3	4	0.25	0.07	>128	>128	>128	>128	>128	>128	64	>128	>128
**M9626**	24	>128	8	48	128	64	24	8	>128	80	20	40	1	0.25	0.095	>128	>128	>128	>128	>128	80	>128	96	>128
**M9643**	40	>128	64	32	>128	>128	32	4	>128	96	16	32	2	0.25	0.155	>128	>128	>128	>128	>128	80	>128	128	>128
**M9647**	10	32	64	40	>128	>128	48	2	>128	>128	32	32	2	0.25	0.155	>128	>128	>128	>128	>128	24	128	80	>128
**M9653**	>128	>128	40	96	128	128	80	1	>128	12	24	10	0.5	0.13	0.188	>128	>128	>128	>128	>128	80	>128	96	>128
**M9654**	20	64	32	24	128	64	24	4	>128	80	24	24	1	0.25	0.375	>128	>128	>128	>128	>128	32.5	128	96	>128
**M9656**	40	64	32	24	128	128	32	4	>128	96	40	24	1	0.25	0.375	>128	>128	>128	>128	>128	40	128	96	>128
**M9660**	40	64	4	24	128	64	40	4	>128	96	20	12	0.5	0.25	0.19	>128	>128	>128	>128	>128	48	128	96	>128
**M9672**	80	>128	8	48	128	128	80	2	>128	64	16	4	0.5	0.5	0.093	>128	>128	>128	>128	>128	96	128	>128	>128
**M9674**	2.5	8	24	32	128	128	40	2	>128	24	8	2	0.25	0.5	0.1875	>128	>128	>128	>128	>128	32	>128	64	>128

Table S2. Lung histopathology findings at 0-6 weeks post infection for ATCC 19977-infected mice receiving 8mg/kg/day dexamethasone until 2 weeks post infection and 12 mg/kg/day dexamethasone thereafter (8+12 regimen). Histopathologic scores for each organ are reported in parentheses after the narrative description.

Weeks post infection	Lung	Spleen	Liver
**0**	Normal (0, 0)	Normal (0, 0)	Normal (0, 0)
**2**	Solitary small AFB focus without granulomatous response in 1 of 2 lungs. Overall structure preserved in both lungs (0, 1)	Normal (0, 0)	Normal (0, 0)
**4**	Tissue invasion of AFB seen in 1 of 2 lungs without granulomatous response. Overall structure preserved in both lungs (0, 2)	Normal (0, 0)	1 of 2 livers with severe steatosis. Second liver normal structure. No AFB organisms seen in either liver (0, 1)
**6**	Diffuse invasion of lung spaces by AFB organisms with purulence in bronchioles but no granulomatous response in 1 of 2 lungs. Second lung normal with no AFB seen. Overall structure preserved in both lungs. (0, 4)	1 of 2 spleens with splenomegaly and areas of scarring. Normal structure in other sample. No discrete AFB seen in any spleen (0, 1)	2 of 2 livers with congestive hepatopathy. No AFB organisms seen in either liver (1, 1)
**7 (death)**	Large AFB+ granuloma in 1 of 2 lungs distorting normal structure. Diffuse AFB tissue invasion in other lung with preserved structure (3, 5)	2 of 2 spleens with splenomegaly with occasional fuchsin-positive subcapsular cells, but no discrete AFB seen in either spleen (2, 2)	2 of 2 livers with steatosis. 1 of 2 livers with multiple AFB+ microabscesses (1, 4)

Disease Models & Mechanisms • Supplementary information
